# S187. SEARCHING FOR BRAIN CO-EXPRESSION MODULES THAT CONTRIBUTE DISPROPORTIONATELY TO THE COMMON POLYGENIC RISK FOR SCHIZOPHRENIA

**DOI:** 10.1093/schbul/sby018.974

**Published:** 2018-04-01

**Authors:** Javier Costas, Mario Paramo, Manuel Arrojo

**Affiliations:** Instituto de Investigacion Sanitaria de Santiago de Compostela-SERGAS

## Abstract

**Background:**

Genomic research has revealed that schizophrenia is a highly polygenic disease. Recent estimates indicate that at least 71% of genomic segments of 1 Mb include one or more risk loci for schizophrenia (Loh et al., Nature Genet 2015). This extremely high polygenicity represents a challenge to decipher the biological basis of schizophrenia, as it is expected that any set of SNPs with enough size will be associated with the disorder. Among the different gene sets available for study (such as those from Gene Ontology, KEGG pathway, Reactome pathways or protein protein interaction datasets), those based on brain co-expression networks represent putative functional relationships in the relevant tissue. The aim of this work was to identify brain co-expression networks that contribute disproportionately to the common polygenic risk for schizophrenia to get more insight on schizophrenia etiopathology.

**Methods:**

We analyzed a case -control dataset consisting of 582 schizophrenia patients from Galicia, NW Spain, and 591 ancestrally matched controls, genotyped with the Illumina PsychArray. Using as discovery sample the summary results from the largest GWAS of schizophrenia to date (Psychiatric Genomics Consortium, SCZ2), we generated polygenic risk scores (PRS) in our sample based on SNPs located at genes belonging to brain co-expression modules determined by the CommonMind Consortium (Fromer et al., Nature Neurosci 2016). PRS were generated using the clumping procedure of PLINK, considering several different thresholds to select SNPs from the discovery sample. In order to test if any specific module increased risk to schizophrenia more than expected by their size, we generated up to 10,000 random permutations of the same number of SNPs, matched by frequency, distance to nearest gene, number of SNPs in LD and gene density, using SNPsnap.

**Results:**

As expected, most modules with enough number of independent SNPs belonging to them showed a significant increase in Nagelkerke’s R2 in our case-control sample after the addition of the module-specific PRS in a logistic regression model. Our permutation strategy revealed that most modules did not show an excess of risk, measured by increase in Nagelkerke’s R2, in comparison to equal number of SNPs with similar characteristics. But one module, M2c from Fromer et al., remained highly significant after multiple tests’ correction. Reactome pathways analysis revealed an over-representation of genes involved in “Neuronal System” and “Axon guidance” among genes from this module. Using the same protocol, we detected that the 84 genes from the neuronal system pathway at this module, representing less than 6% of the genes from the module, explained a higher level of risk than expected. “Voltage-gated Potassium channels” and “Neurexins and neuroligins” are overrepresented among the Neuronal System genes from module M2c.

**Discussion:**

Here, we show that, in spite of the high polygenicity of schizophrenia, it is possible to identify gene sets contributing disproportionately to total risk, as it was the case for the M2c module from Fromer et al. These authors have previously reported that the M2c module was enriched in GWAS signals, as well as CNVs and rare variants associated with schizophrenia. Therefore, this module shows a disproportionately contribution to schizophrenia risk.

Study supported by Grant PI14/01020 from Instituto de Salud Carlos III, Ministry of Health, Spanish Government.

